# Supplementary material for: Predicting visual function by interpreting a neuronal wiring diagram
Source: Nature. 2024 Oct 2;634(8032):113–23. doi: 10.1038/s41586-024-07953-5 (PMC11446822; doi:10.1038/s41586-024-07953-5)
Supplement: Supplementary file 6 — CRF and ERF predictions for individual TmY4 and TmY9 cells. Analogous to Supplementary Data 3, but for TmY target types. Shown are the top four monosynaptic pathways, the strongest pathway passing through each of the top ten intermediary types (ranking from Extended Data Fig. 7), and the trisynaptic pathway Tm1–TmY–Dm3–TmY (see the section entitled Prediction of spatial normalization). [file 41586_2024_7953_MOESM6_ESM.zip › DataS4/TmY9qΓèÑ/720575940625217978.pdf]

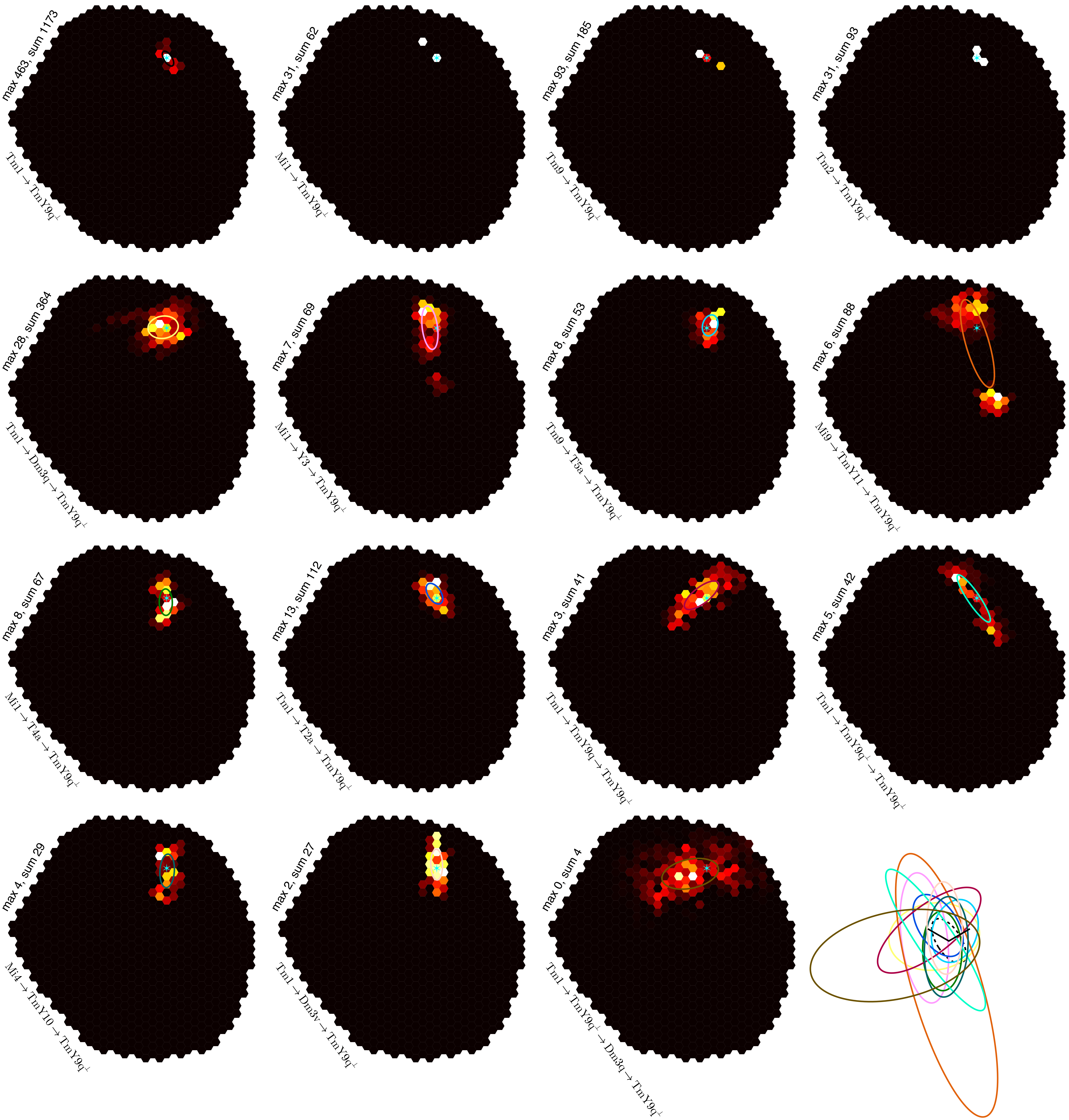
max 463, sum 1173
Tm1 → TmY9q⊥
max 31, sum 62
Mi1 → TmY9q⊥
max 93, sum 185
Tm9 → TmY9q⊥
max 31, sum 93
Tm2 → TmY9q⊥
max 28, sum 364
Tm1 → Dm3q → TmY9q⊥
max 7, sum 69
Mi1 → Y3 → TmY9q⊥
max 8, sum 53
Tm9 → T5a → TmY9q⊥
max 6, sum 88
Mi9 → TmY11 → TmY9q⊥
max 8, sum 67
Mi1 → T4a → TmY9q⊥
max 13, sum 112
Tm1 → T2a → TmY9q⊥
max 3, sum 41
Tm1 → TmY9q → TmY9q⊥
max 5, sum 42
Tm1 → TmY9q⊥ → TmY9q⊥
max 4, sum 29
Mi4 → TmY10 → TmY9q⊥
max 2, sum 27
Tm1 → Dm3v → TmY9q⊥
max 0, sum 4
Tm1 → TmY9q⊥ → Dm3q → TmY9q⊥
